# Meta-analysis of randomized controlled trials of electronic health interventions to reduce medication errors

**DOI:** 10.1038/s41746-025-02127-7

**Published:** 2025-12-17

**Authors:** Michelle Natasha Colin, Salma Tri Octaviany, Michelle Darmawan, Joseph Fide Anggi, Clara Fernanda Kusuma, Angga Prawira Kautsar

**Affiliations:** 1 Faculty of Pharmacy, Universitas Padjadjaran, Sumedang, West Java Indonesia; 2 Center of Excellence in Higher Education for Pharmaceutical Care Innovation, Universitas Padjadjaran, Sumedang, West Java Indonesia; 3 Department of Pharmaceutics and Pharmaceutical Technology, Universitas Padjadjaran, Sumedang, West Java Indonesia; 4 Unit of Global Health, Department of Health Sciences, University of Groningen (RUG)/University Medical Center Groningen (UMCG), Groningen, The Netherlands

**Keywords:** Health policy, Public health

## Abstract

Medication errors, both potential and actual errors, can pose a significant safety concern in healthcare. Potential errors refer to mistakes detected and prevented (either manually or electronically), while actual errors include both harmless and harmful events, such as adverse drug events (ADEs). Electronic interventions, particularly computerized decision-support systems (CDS), aim to reduce medication errors and thus enhance patient safety. Our study conducts systematic reviews and a meta-analysis on the effectiveness of electronic interventions in decreasing medication errors compared to usual care. Randomized controlled trials (RCTs) articles from EBSCO, Embase, PubMed, and Web of Science databases were systematically screened and selected. The analysis included 12 studies, finalized by nine after addressing heterogeneity. The results demonstrated that electronic interventions were associated with a 15% reduction in risk of medication errors (RR = 0.85; 95% CI: 0.77–0.94). Subgroup analysis showed that CDS interventions were particularly effective in our study. The findings provide evidence for the potential benefit of integrating electronic systems to enhance medication safety. Further research is necessary to validate these outcomes across a range of settings.

## Introduction

Medication errors can pose a significant threat to patients, potentially leading to severe adverse effects, prolonged hospital stays, increased healthcare costs, and even fatalities^1^. These incidents can be classified into several types: potential errors, which are identified and prevented before reaching the patient either manually or electronically; unidentified errors that do not cause harm; and adverse drug events (ADEs). Such mistakes can occur at any stage of the medication process, resulting in serious consequences for both patients and healthcare providers^2^. Errors arising from inappropriate medication use have been linked to increased morbidity and mortality, while also compromising trust in healthcare systems and exposing providers to legal and regulatory risks^3–5^. These occurrences can take place in various settings, including hospitals, clinics, pharmacies, and private drug outlets^6^, with hospitals^7^ being the most frequent site due to the complexity and volume of care delivered. Given the clinical and systemic implications for both patients and providers, interdisciplinary collaboration—such as between prescribers, pharmacists, and clinical IT teams—has been shown to be effective in reducing medication errors^8^. Unsafe care affects ~1 in 10 patients and leads to over 3 million deaths annually, underscoring its global prevalence. This impact was particularly severe in low- to middle-income countries, where as many as 4 in 100 individuals may die from unsafe care^6^. Moreover, more than 50% of these incidents (1 in every 20 patients) were preventable, with half attributed to medication-related issues^8,9^. Furthermore, drug administration errors, as reported in a 2016 study by NORC at the University of Chicago, range from 8% to 25%. Therefore, exploring effective interventions and strategies to address this issue is crucial for mitigating medication errors and enhancing patient safety^10^.

Electronic interventions, such as computerized decision-support systems (CDS), have been increasingly adopted to reduce medication errors and enhance patient safety by providing real-time clinical guidance and alerts. Supporting technologies—including electronic health records (EHR), barcoding, standardized units, weight-based dosing, and pharmacist-led medication reviews^11^—have reduced medication errors^12–16^. In particular, implementing electronic medication administration records (eMAR) and computerized physician order entry (CPOE) systems, such as barcode medication administration (BCMA), has played a crucial role in reducing administration errors^17^. A study involving Indonesia’s National Health Insurance population found a statistically significant association between prescribing errors and service quality, with common issues such as missing patient age (35.2%), address (8%), weight (4.4%), and physician’s name (0.8%) identified as key contributors^18^. These findings highlight the potential benefit of integrating electronic systems in reducing medication errors and improving healthcare service quality.

While previous systematic reviews have examined the role of electronic interventions in reducing medication errors, many have focused on observational studies or a single type of intervention, limiting the generalizability of their findings^19–21^. Scoping and narrative reviews, on the other hand, have provided valuable context by mapping the range of available interventions and highlighting emerging approaches. However, they often lack the methodological rigor required to quantify effectiveness^22^. Moreover, these reviews often lack comparative analyses of different electronic strategies or do not assess their effectiveness across diverse clinical settings. Distinguishing from prior research^19–21^, our study adopts a novel approach by exclusively focusing on RCTs and conducting a meta-analysis to evaluate the effectiveness of electronic interventions—including CDS and electronic alerts. The primary objective was to evaluate the impact of electronic interventions on medication errors across varied clinical settings, while the secondary objective was to classify the types of electronic interventions used. This approach aims to generate evidence-based insights to guide future healthcare practice and policy toward improved patient safety.

## Results

### Study selection

**Fig. 1:**
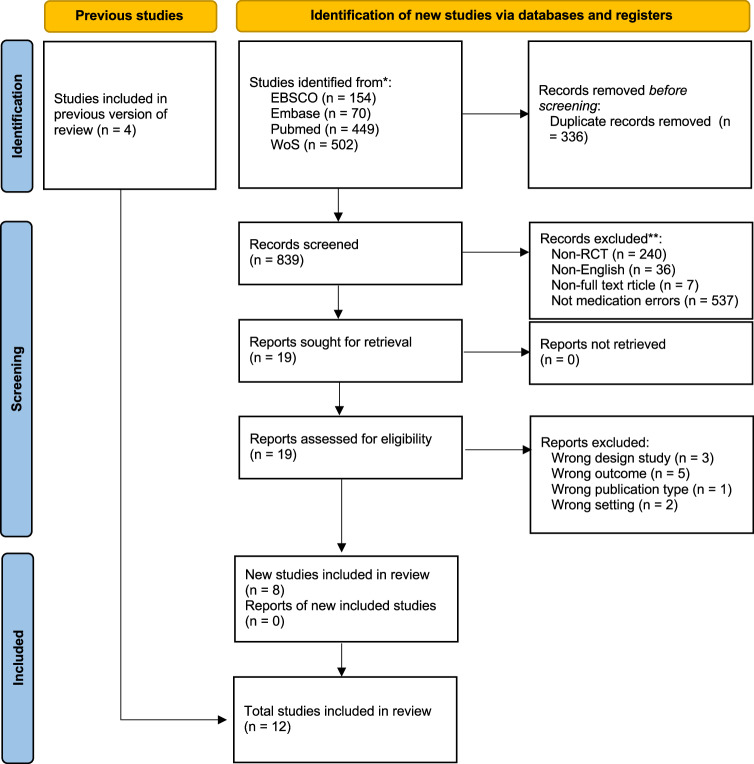
Preferred reporting items for systematic reviews and meta-analyses (PRISMA) diagram.

A thorough search across major databases identified 1175 studies during the selection process. After removing 336 duplicate entries, 839 unique studies proceeded systematic screening (Fig. 1).

Following the screening process, 19 studies were retrieved and assessed for eligibility. Eight new studies were deemed eligible for inclusion in the review. In addition, we incorporated four studies from a previous systematic review and meta-analysis study by Roumeliotis et al.^19^ that were not captured by our search. The cumulative result was 12^13,23–33^^,^ forming the basis for the subsequent meta-analysis.

### Characteristics of included studies

The included studies in this review varied in terms of geographic locations and study designs, offering valuable insights into preventing medication errors. Approximately 50% of studies were conducted in the United States (USA), with others originating in various European locations. Medication errors were predominantly observed in male subjects and were frequently reported among individuals aged 50–80. Three types of interventions were identified in the studies reviewed, including CDS, electronic alerts, and e-learning provided to physicians. The extracted data show that electronic interventions, such as electronic alerts and CDS, improve patient safety and reduce medication errors better than usual care (Table 1). Further population data in the included studies have been extracted in Supplementary Table 8. Population Characteristics.

**Table 1:** Studies characteristic

No	Author (year)	Country	Intervention	Comparator	Design study	Objectives	Outcome	Additional outcome
1	Berdot et al.^13^	Paris, France	CDS	Usual care	RCT	To evaluate the effect of a commercial BCMA system on technician medication errors during the dispensing stage and evaluate integration process barriers.	Dispensing error rates	Lessons from this study will be revealed through the identification of barriers encountered
2	Le Meur et al.^24^	Limoges, France	CDS	Fixed dose group, in which mycophenolate mofetil dose modifications based on clinical experience were permitted, and physician were not given access to mycophenolic acid under area curve data	RCT (12-month, multicenter, open-label study with sequential enrollment)	Reductions in treatment failure.	Treatment failure	To compare the frequency of adverse events and the severity of acute rejection that is biopsy-proven and clinically suspected in the two groups
3	Tamblyn et al.^32^	Quebec, Canada	CDS	On-Demand Decision Support	A single-blind cluster RCT	To evaluate the advantages of on-demand versus customizable computer-triggered drug decision support in terms of reducing the incidence of prescription errors.	The prevalence of prescription issues at the end of the observation period	The number of alerts that were observed
4	Newton et al.^19^	Atlanta, USA	CDS	A standard paper-form insulin infusion algorithm	RCT	To assess the effectiveness and safety of continuous insulin infusion in a medical intensive care unit using the standard paper-form algorithm compared to the computer-guided method.	To compare the mean daily blood glucose concentration between treatment groups to assess variations in glycemic control.	Variations in the incidence of hypoglycemia episodes, the amount of insulin administered, the length of hospital and ICU stays, the number of hyperglycemic episodes, and the mortality rate between groups
5	Strom et al.^33^	California, USA	Electronic alert	Standard practice	RCT	To investigate the efficacy of a CPOE prescribing warning that is almost “hardstop.”	Concurrent prescriptions for trimethoprim-sulfamethoxazole and warfarin	
6	Dumont & Bourguignon^26^	Virginia, USA	CDS	Paper protocol	Prospective RCT	To investigate the impact of utilizing an automated insulin dosage calculator to support critically ill cardiac patients in managing their glycemic control.	Nurse satisfaction scores, blood glucose measurement variability, and the proportion of measurements in the target range	
7	McCoy et al.^23^	Nashville, USA	CDS	Common services for clinical pharmacy and clinical decision support	RCT	To enhances prescription practices in the context of acute kidney injury (AKI), but there is still room for improvement in patient safety and to evaluate the possibility that pharmacist surveillance of AKI patients could identify and stop drug errors that are not fixed by automated treatments.	Blinded examination of the duration until a provider modifies or stops providing specific nephrotoxic or renally cleared medicine, as well as potential adverse drug events (pADEs) and ADEs	
8	Geurts et al.^27^	Rotterdam, Netherlands	CDS	Usual care	RCT	Comparing the management of children with acute gastroenteritis at risk for dehydration.	Nurses’ adherence to the recommendations	To ascertain the relationship between the degree of dehydration and weight change
9	Ashburner et al.^28^	Boston, USA	Electronic alert	The intervention’s impact was not statistically significant in any subgroup and for those with prior anticoagulation	RCT	To investigate whether the percentage of ambulatory individuals who receive an oral anticoagulant prescription increases when physicians receive electronic notifications.	The amount of patients prescribed oral anticoagulants after three months in the notification arm versus the usual care arm.	Survey-based justifications for not recommending oral anticoagulants and the steps that physicians planned to take in response to the warning
10	van Stiphout et al.^29^	Rotterdam, Netherlands	E-learning given to physicians	Usual approach	Two-arm cluster RCT	Demonstrating the superiority of the task analysis-based educational intervention.	The proportion of medication discrepancies per physician	The amount of patients per physician who have had at least one drug drug interaction that was overlooked and could have resulted in a clinically adverse drug event
11	Adusumalli et al.^30^	Pennsylvania, USA	Electronic Alert	Cardiologists receiving standard treatment did not receive any further interventions	Three-arm cluster RCT	Using an interrupted alert that required action before the clinician could continue using the electronic health record, the study compared the use of guidelines-directed statin prescribing among cardiologists who were randomized to usual care.	The shift in the proportion of individuals who are administered statin medication at a dosage that complies with evidence-based recommendations	The shift in the proportion of qualified patients who receive a statin prescription at any dose
12	Westbrook et al.^31^	New South Wales, Australia	CDS	Hand-written on paper charts	SWCRCT	Comparison of the prescription errors that occured during the time of control.	This study evaluates an electronic medication management (eMM) system’s ability to decrease prescribing errors and their potential and actual harm throughout the short (first 70 days of eMM) and long (one year) time periods	

### Risk of bias analysis

**Fig. 2:**
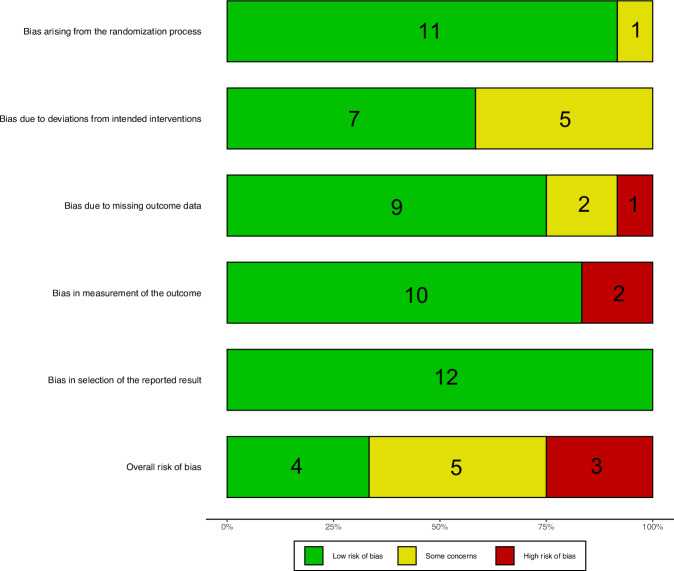
Risk of Bias Analysis. RoB analysis is to examine the internal validity of included studies using the relevant domain-based RoB assessment tool and present the results of this assessment in a graphical format.

The Risk of Bias (RoB) analysis results indicate a 25% high risk, as shown in the graph and summary in Fig. 2. Each author independently assessed this RoB. In summary, six (6/12, 50%) studies were evaluated as having a low risk across all domains, four (4/12, 33%) studies were deemed to have some concerns in at least one domain, and two (2/12, 17%) studies were classified as high risk in at least one domain. Supplementary Fig. 3 presents the RoB per domain.

### Meta-analysis of medication error results

**Fig. 3:**
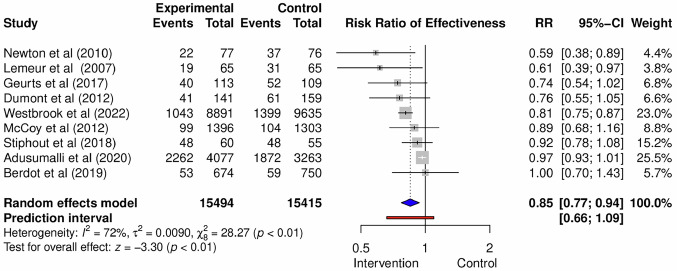
Forest Plot. Forest plot visually summarizes the findings of multiple studies, allowing for easy comparison of their results. The key components of the forest plot include individual study results, effect size, confidence intervals, study weight, overall effect, and line of no effect.

After pooling the effect sizes of the data from 12 included studies, the forest plot (Fig. S6) indicates a relative or risk ratio (RR) of 0.70 (95% confidence interval (CI): 0.51–0.97). We recognized high heterogeneity in the data, with a between-study heterogeneity variance of *τ*^2^ 0.24 (95% CI: 0.11–0.73) and an *I*^2^ value of 96% (95% CI: 94.40–97.20). To address this high heterogeneity, we conducted additional analyses—such as outlier removal, influence diagnostics, and Graphical Display of Study Heterogeneity (GOSH) plot inspection (Supplementary)—which led to the exclusion of three influential studies (33, 37, 40). After removing these studies and reapplying the analysis, the final version of forest plots is shown in Fig. 3. The RR for the remaining nine studies was 0.85 (95% CI: 0.70–0.94). The prediction interval for the post-removal studies (95% CI: 0.66–1.09) was getting narrower than before-removal studies (95% CI: 0.43–1.15). The data were displayed in tabular form to provide additional information (Fig. 3, Forest Plot).

We conducted two types of subgroup analyses, which were by intervention and by outcome. For intervention subgroups, seven studies evaluated CDS, one assesed e-alert, and the last one investigated an e-learning intervention. Meanwhile for the outcome subgroups, three studies evaluated inappropriate prescriptions, four evaluated medication erros, and two assessed ADEs. We conducted subgroup analysis that the forest plot of subgroup intervention reveals the following RR: electronic alert (0.97, 95% CI: 0.93–1.01), e-learning (0.92, 95% CI: 0.78–1.08), and CDS (0.80, 95% CI: 0.75–0.86) (Fig. S3). The RR of subgroup outcome for inappropriate prescription/drug was 0.95 (95% CI: 0.89–1.02), for medication error was 0.81 (95% CI: 0.75 −0.86), and for ADE/reaction was 0.77 (95% CI: 0.55–1.10) (Fig. S4).

### Sensitivity analysis

Sensitivity analysis yielded an adjusted estimate of RR (0.87, 95% CI: 0.79–0.95) and an unadjusted estimate (0.85, 95% CI: 0.77–0.94) with a significance level for the test of residual selection bias set at 0.1. The *p* value obtained from this sensitivity analysis was 0.0023 for the adjusted overall treatment effect, 0.0010 for the unadjusted effect, and 0.13 for the test of residual selection bias. More detailed data were provided in Supplementary Table 11 and 12. Publication and reporting biases analysis.

**Fig. 4:**
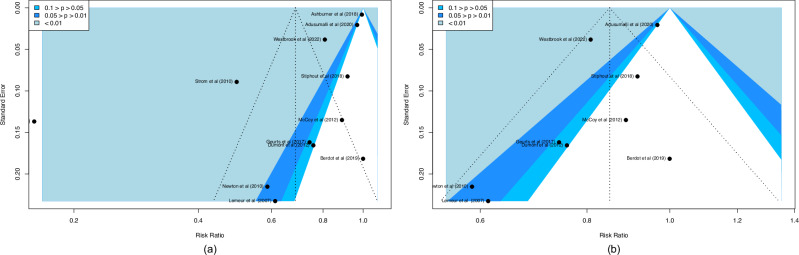
Funnel plot. Funnel plot provides a visual representation of the relationship between the effect sizes of individual studies and their precision. **a** Funnel plot before study removal. **b** Funnel plot after study removal.

When all studies were included in the funnel plot (Fig. 4), the outcome displayed a non-symmetrical distribution, indicating that publication bias affected the study. However, after removing three papers based on the heterogeneity test’s suggestions, the updated plot showed a balanced distribution of the included studies, indicating that publication bias probably did not affect the results.

**Fig. 5:**
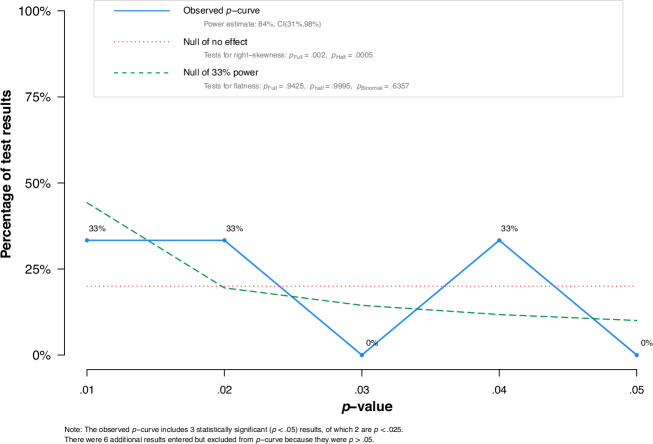
P-curve analysis. P-curve analysis examines the pattern of statistically significant *p* value reported in studies.

Our meta-analysis data were assessed to determine the presence of a genuine effect and to estimate its size using P-curve analysis (Fig. 5), which focuses on *p* value as the primary contributor to publication bias. Tests for right-skewness resulted in values for Pfull (*P* = 0.002) and Phalf (*P* < 0.001), while the tests for flatness resulted in values for Pfull (*P* = 0.942), Phalf (*P* = 0.999), and Pbinomial (*P* = 0.636).

## Discussion

This meta-analysis found that electronic interventions—particularly CDS—were associated with a 15% reduction in the risk of medication errors compared to usual care (RR = 0.85, 95% CI: 0.70–0.94). These findings support the potential of electronic systems to enhance patient safety by reducing human error in medication processes. This result aligns with prior reviews, including Roumeliotis et al. (2019), who reported risk reductions with electronic prescribing strategies, although with a broader inclusion of study designs and higher heterogeneity (*I*^2^ = 98%). In contrast, the present study focused exclusively on RCTs, offering more rigorous evidence and greater internal validity.

Electronic interventions, particularly CDS and CPOE, have been reported to improve specific outcomes compared to traditional methods such as training programs, policy changes, or manual checks. These technologies enhance consistency, reduce human error, and improve efficiency by providing real-time decision support. While human oversight remains essential for complex clinical judgments, automation may reduce errors related to fatigue or workload by standardizing critical processes. As such, a hybrid approach combining human expertise with electronic safeguards may serve as a complementary safeguard against medication errors. However, an integrated approach combining automation and human oversight may be beneficial, leveraging the efficiency benefits of technology while retaining human flexibility for complex situations^34^.

The included studies were predominantly conducted in high-income countries such as USA and European nations, where advanced health information infrastructure supports the adoption of electronic health technologies. These settings have enabled broader implementation of such interventions^35^. Several studies noted a higher prevalence of medication errors among older adults (aged 50–80), likely due to polypharmacy, comorbidities, and age-related physiological changes^36^. In Australia, for instance, nurses reported positive perceptions of automated dispensing systems, which contributed to reduced medication errors^19^.

Comparison with previous meta-analyses^19,20^ highlights the value of focusing on RCTs. While other reviews, such as those by Gates et al. (2020) and Osmani et al. (2023), included observational or pre-post study designs, our analysis provides a more rigorous estimate of intervention effectiveness^21,37^. While prior studies reported comparable results to ours, their findings may have been influenced by selection bias or the absence of control groups attributable to different study designs. By contrast, our RCT-only approach improves internal validity and increases confidence in causality^38^.

High heterogeneity in the outcomes makes drawing solid conclusions challenging^39^. Heterogeneity was initially substantial, prompting outlier analysis and sensitivity testing. After removing three influential studies—including one with an extremely low RR (0.16)^32^—the heterogeneity statistics improved (*I²* decreased, confidence intervals narrowed), although the RR slightly increased. This adjustment was associated with improved statistical precision and reduced variability, as indicated by the narrower prediction interval. The overlap between confidence and prediction intervals suggested consistent intervention effects across similar populations^38,40^.

Subgroup analysis showed variability in effect size based on the type of intervention. CDS consistently demonstrated a lower RR compared to other strategies, which may be attributed to its capacity to support CDS and reduce cognitive workload^41^. Studies by Chen et al. (2024), Dahmke et al. (2024), and Miao et al. (2025) further underscore the benefits of CDS in enhancing workflow efficiency and reducing errors^42–44^. Subgroup analysis by outcome type also revealed that electronic interventions reduced ADE, although the wide CI suggests some uncertainty due to smaller sample sizes. ADEs are harmful events associated with medication that are critical for patient care, quality improvement, drug safety research, and post-marketing surveillance ^45^. It is routinely documented by providers in clinical records to inform patient care^46^. In contrast, while these studies have a higher RR, they exhibited more precise estimates and lower heterogeneity, likely to resulting from standardized outcome definitions, consistent reporting practices, uniformity in measurement tools, and comparable study designs, methodologies, and statistical approaches^47,48^.

RoB analysis revealed some concerns, particularly related to deviations from intended interventions in hospital-based trials. Such deviations may compromise randomization, introducing potential confounding^49^. Additional risks stemmed from missing outcome data and inconsistent measurement methods, reflecting the diversity in intervention types and reported endpoints. However, the reporting bias was considered low due to the consistent outcome reporting across studies.

Most included studies employed independent evaluators for outcome measurement. In cases where self-reporting was used, it served a supplementary role, ensuring that objective data collection remained the primary basis for analysis. This methodological consistency supports the internal validity of the findings^13,23–33^.

Despite using various electronic strategies across studies—ranging from CDS and CPOE to barcoding and alerts—all interventions targeted aspects of the medication-use process. These differences in scope and endpoints led to heterogeneity while also highlighting the possible additive effects of multifaceted strategies. By incorporating diverse approaches, future implementations can tailor interventions to local needs and address complex sources of medication error^50^.

After removing outliers, funnel plots appeared more symmetrical, indicating improved precision in effect estimation. The P-curve also showed right-skewed significance patterns and flatness test results consistent with evidential value, supporting the conclusion that the observed effect was unlikely to be due to selective reporting. Collectively, P-curve analysis findings show evidential value and an actual non-zero effect^48^. These findings provide evidence for the validity of meta-analysis results by confirming that the observed reduction in medication errors was supported by genuine evidential value.

We acknowledge several limitations in our study, a key one being the relatively small number of included studies. Although we addressed this by performing a sensitivity analysis to assess the findings robustness based on primary data analyses, variability in the results was observed when certain studies were excluded or different methods were applied^51^. This suggests that the findings may be influenced by the specific studies and methodological selections, warranting caution in interpreting the results. Such variability also emphasizes the need to include a broader range of studies and apply consistent, validated methodological approaches. Additionally, to mitigate potential publication bias, it was crucial to emphasize high-quality research, publish valid trials regardless of their outcomes, and ensure peer reviewers disclose any conflicts of interest. Therefore, we conducted literature reviews to address this limitation. Involving various electronic interventions in the analysis complicates the understanding of how each technology enhances healthcare, as they address different stages of the medication process. Grouping them together may obscure specific mechanisms through which each intervention could improve patient safety. Nevertheless, the pooled evidence remain provides relevant insights for clinical and policy implementation. Medication errors, although preventable in many cases^52^, remain a major cause of patient harm and mortality. Integrating electronic systems such as CDS can aid healthcare providers in minimizing human error and enhancing patient safety. To translate these findings into practice, healthcare institutions may consider prioritizing the adoption of electronic interventions within standard care workflows, supported by adequate training for clinical staff. Moreover, promoting a culture of patient safety and non-punitive error reporting is considered essential. Patient education should also be incorporated as part of a comprehensive approach to reducing medication errors^14^. These findings underscore the need for healthcare administrators and policymakers to prioritize the implementation of electronic decision-support tools, particularly in settings with existing technological infrastructure. To address current limitations, future research should prioritize pragmatic trials and real-world studies that generate robust evidence while maintaining ecological validity. Designing studies with larger sample sizes, longer follow-up durations, and standardized outcome measures may enhance comparability and reduce bias. Comparative effectiveness research is needed to evaluate various types of electronic interventions—such as CDS, e-prescribing, and medication administration systems—for different types of medication errors. Furthermore, future studies should investigate the cost-effectiveness, sustainability, and scalability of these interventions, particularly in resource-constrained settings. Investigating implementation barriers—including technological infrastructure, organizational readiness, and healthcare professionals’ acceptance—is essential for optimizing uptake. Leveraging real-world data and incorporating artificial intelligence tools may offer more granular and practice-relevant insights. Finally, integrating mixed-methods approaches can deepen understanding of how such interventions affect clinical workflows and promote a culture of medication safety.

The findings of this review indicated that the implementation of electronic interventions in hospital settings was associated with an approximate 15% reduction in the risk of medication errors, although the effect size varied across studies. Among the various types of interventions, CDS systems emerged as the most effective strategy in this analysis, evidenced by the lowest RR and multiple supporting studies. Although electronic interventions are being increasingly adopted in practice, the limited number of RCTs assessing their effectiveness limits the conclusiveness of current evidence and reliability of the current evidence. Conducting RCTs in this context presents multiple challenges, including ethical issues, logistical difficulties, and the complexity of implementing electronic systems across multifaceted healthcare environments. Unlike drug trials, electronic health interventions involve numerous interacting components, making strict randomization and control more difficult to achieve. Consequently, existing evidence was derived from observational studies, which are typically more prone to bias and confounding factors.

## Methods

### Eligibility criteria

This systematic review was conducted in accordance with the PRISMA^53^ and Cochrane guidelines^54^ to ensure methodological rigor and transparency. A detailed PRISMA checklist and the full search strategy are provided in the supplementary materials. A detailed protocol outlining each review step was followed to enhance methodological transparency and reproducibility. The type and design of this study align with established standards for systematic reviews^40,53,55^. The eligibility criteria for the research studies were determined using the Population, Intervention, Comparator, and Outcomes (PICO) framework. PICO framework has been utilized throughout the review process to ensure a systematic and clear approach to formulating our research questions and maintain a consistent understanding of the study objectives and methodologies among the researchers involved. In cases where researchers lack agreement, we have established a consensus approach to ensure that all perspectives are considered and that our findings are thoroughly examined and supported by the available evidence. Studies were included in this systematic review if they met a series of eligibility criteria structured according to the PICO framework. The defined population (P) consisted of patients receiving medication prescriptions in a hospital setting. The intervention (I) of interest was electronic prescribing, such as CPOE or CDS, compared to a comparator (C) of manual, paper-based prescribing or usual care without electronic intervention. The required outcome (O) was the reporting of quantitative data on the incidence of medication errors. In addition to the PICO criteria, studies were required to be RCTs, published in English, and available as full-text, peer-reviewed articles. Studies were excluded from this review if they were non-human studies, had a study design other than an RCT (such as reviews, editorials, letters, or conference abstracts), were not available in full-text, or reported incomplete outcome data for quantitative synthesis.

### Information sources

The following electronic databases were searched for relevant literature: EBSCO, Embase, PubMed, and Web of Science (WoS). In addition to the database search, the reference lists of all included studies and relevant systematic reviews were manually screened to identify additional eligible articles.

### Search strategy

Search phrases were developed based on the PICO framework and relevant Medical Subject Headings (MeSH) terms. The search strategies incorporated both free-text terms and MeSH, particularly in the PubMed database, to enhance retrieval sensitivity and coverage. We conducted search in May 2023, with no restrictions or filters applied at the initial stage. To track updates, email notifications from the searched databases were activated from the initial search date until the manuscript submission date. A detailed example of the search terms for each database was provided in Supplementary Tables 2–5. The search strategies were adapted for each database with the assistance of an information specialist to ensure comprehensive coverage. In each database, we applied a structured search strategy using combinations of free-text keywords and controlled vocabulary (e.g., MeSH terms), connected through Boolean operators (AND/OR).

### Selection process

The study rigor was enhanced by employing the multi-reviewer approach to analyze the studies. STO and MNC saved the search results in a standardized format for future accessibility. Duplicate records were first removed using Mendeley Desktop. The remaining studies were then imported into Rayyan for title and abstract screening. Within the app, we manually applied filters to exclude non-human studies and non-RCTs by tagging and excluding records based on article type and subject population metadata. Each screening step was performed independently by two reviewers (MCN and STO), with conflicts resolved through consensus or by a third reviewer (APK). The selection process was illustrated in the PRISMA flowchart (Fig. 1)^53^. The supplemental materials include the PRISMA 2020 Checklist and Abstract Checklist in Supplementary Table 6-7 PRISMA Diagram.

### Data collection process

Four researchers (MCN, STO, MD, and JFA) independently reviewed and gathered data from eligible studies. Then, four researchers extracted the data collected on general and population parameters, such as author and publication year, study design, location, number of study participants, age and gender, and intervention and control groups. Next, four researchers also gathered, in an analogous manner, outcome information, including study and patient characteristics (country, year of research, patients visiting all healthcare facilities, both outpatient and hospitalized, all diseases), and RR of intervention in reducing medication errors or inappropriate rate numbers. If necessary, APK was consulted to make the final decision. The next step involves cross-checking the report for correctness to see if there were any differences. All extracted data were standardized in a tabular format and available in supplemental materials. Data extraction forms were piloted and standardized prior to full extraction to ensure consistency across reviewers.

### Data items

The primary outcome of electronic intervention was to reduce medication errors. Preventing ADEs was one strategy to reduce the number of medication errors and drug misuse^39^. Based on eligibility criteria, participants were divided into two groups: intervention and control. Secondary outcomes pertain to the type and number of medication errors in hospital settings. We obtained the results by selecting the outcome definition that was prevalent across studies and was an important finding. We specifically sought events that qualified as medication errors to ensure that all results matched the outcome criteria. The included studies were further grouped into subgroups according to both the type of electronic intervention used (Fig. S3) and the outcomes (Fig. S4). Interventions were divided into three categories: (1) electronic alert, (2) e-learning, and (3) CDS. The outcomes were also divided into three groups: (1) inappropriate prescription/drug, (2) medication errors, and (3) ADE/reaction. Exploratory analyses were then performed by pooling studies within the same groups.

### RoB assessment

Version 2 of the Cochrane RoB tool for randomized trials (RoB 2.0) was employed by MNC, STO, MD, JFA, and CFK to assess the RoB in clinical trials^38^. This risk assessment encompasses five areas: bias arising from the randomization process, bias due to deviations from intended interventions, bias due to missing outcome data, bias in the measurement of the outcome, and bias in the selection of the reported result. The bias was classified into three categories: high, some concerns, and low. The highest RoB level in each of the evaluated domains was used to calculate the overall risks for each study. Any issues that arose were resolved through discussions. The RoB assessments were double-checked by a second reviewer for consistency, and justifications for judgments were documented to ensure traceability^56^.

### Effect measures

We utilized RR to evaluate the effectiveness of electronic systems in reducing medication errors (a binary outcome). This metric compares two risks by dividing the risk in the intervention group and the risk in the control group^49^. A less than one RR indicates that the electronic system reduces the risk of medication errors, signifying an improved outcome. Conversely, more than one RR suggests an increased risk of medication errors in the intervention group. The RR of one indicates no difference in risk between the intervention and control groups^57,58^. Furthermore, we can convert the risk value to a percentage of efficacy or effectiveness of the intervention using the formula: Effectiveness = 1–RR. This conversion provides a straightforward understanding of the intervention’s impact^59^. Additionally, 95% CI, and heterogeneity statistics were provided for each result to assess the reliability and consistency of the findings.

### Data synthesis and analysis

The present investigation employed a thorough extraction methodology to detect occurrences of medication errors in both the intervention and control groups. The errors were systematically classified according to the type of intervention and recorded in the preceding data table. The method’s final step involved categorizing eligible studies based on the availability of outcome data and entering these categories into a standardized table. We also displayed all study and participant characteristics in a tabular format. Given the variety of participant characteristics, such as age, gender, number of patients, number of prescriptions, number of physicians, number of medications, and number of care units, we replaced the earlier DerSimonian-Laird estimator with a constrained maximum likelihood random-effects estimator, in line with recent recommendations^47,60^. This choice was based on recent methodological recommendations favoring greater accuracy in small-sample meta-analyses and high-heterogeneity contexts. A forest plot was subsequently created to examine the combined effect sizes of the results, with outcomes arranged by study name. We assessed heterogeneity between studies using τ² and *I*^2^ statistics. Specifically, *I*^2^ values of 25% indicate a low degree of inconsistency, 25%–50% indicate moderate inconsistency, and >50% indicate high inconsistency. Further heterogeneity analyses will be needed when the results show high inconsistency, including basic outlier removal, influence diagnostics, and GOSH plot^47^.

The 95% CI around the τ² and *I²* values was calculated to assess our confidence in these measurements. The width of the CIs provides information about the accuracy of the study’s effect size estimate, where a narrower interval suggests greater accuracy and a broader interval indicates more uncertainty due to larger standard errors^24^. While CI describes the true effect size within a specific confidence level (e.g., 95%), the prediction interval reflects the likely range of effects for new studies from the same population.

We performed subgroup analysis to examine the differences between the intervention methods and analyzed how effect sizes varied across subgroups^47^. We conducted a sensitivity analysis to assess the robustness and reliability of the synthesized results. A Copas selection model analysis was used in this analysis to evaluate the stability of the study outcomes^60^. If the *p* value for the residual selection bias test (p.rsb) is less than or equal to 0.1, the null hypothesis of no residual selection bias is rejected. On the contrary, if the *p* value is >0.1, the null hypothesis cannot be rejected, suggesting no strong evidence of residual selection bias at the selected significance level^47^. All analyses and graphical representations included in this study were produced utilizing R-Studio version 2022.22.02, in conjunction with the dmetar package^47^.

### Reporting bias assessment

To identify publication bias, we assessed the small-study effect and *P-*curve analysis. Funnel plots are commonly used to examine small-study effects. Contour-enhanced funnel plots help determine how asymmetry patterns relate to statistical significance. In these plots, the *y* axis was typically reversed so that higher values correspond to smaller standard errors. Data points should form an approximately symmetric, upside-down funnel if no publication bias is present^55^. We also performed *P-*curve analysis, which emphasizes *p* value as the primary source of publication bias. The P-curve technique assumes that the shape of the *p* value distribution is influenced by sample size and the true effect size. *P-*curve analysis consists of two tests, a test for right-skewness and a test for flatness^60^. A right-skewed P-curve suggests the presence of a true effect, whereas a left-skewed p-curve points to publication bias or p-hacking. The left skew would occur due to a tendency to obtain a *p* value just below 0.05, even when there is no real effect, indicated by an increased frequency of results with a marginally significant *p* value^61^.

## Supplementary information

Supplementary Information

## Data Availability

All data are publicly available in the cited studies. The code is available upon reasonable request from the corresponding author.

## References

[1] Doshmangir, L., Ahsani-Estahbanati, E. & Gordeev, V. S. Interventions to reduce the incidence of medical error and its financial burden in health care systems: a systematic review of systematic reviews. *Front. Med.* **9**, 875426 (2022). 10.3389/fmed.2022.875426 PMC9363709 35966854

[2] Velo, G. P. & Minuz, P. Medication errors: prescribing faults and prescription errors. *Br. J. Clin. Pharmacol.* **67**, 624–628 (2009). 19594530 10.1111/j.1365-2125.2009.03425.x PMC2723200

[3] Phillips, D. P. & Bredder, C. C. Morbidity and mortality from medical errors: an increasingly serious public health problem. *Annu Rev. Public Health* **23**, 135–150 (2003). 10.1146/annurev.publhealth.23.100201.133505 11910058

[4] Brown, E. Switching clinics: patient autonomy over the course of their careers in consumer medicine. *J. Health Soc. Behav.* **64**, 228–242 (2023). 36843416 10.1177/00221465231154956

[5] Wittich, C. M., Burkle, C. M. & Lanier, W. L. Medication errors: an overview for clinicians. *Mayo Clin. Proc.* **89**, 1116–1125 (2014). 24981217 10.1016/j.mayocp.2014.05.007

[6] Wulandari, L. P. L. et al. Prevalence and determinants of inappropriate antibiotic dispensing at private drug retail outlets in urban and rural areas of Indonesia: a mixed methods study. *BMJ Glob. Health* **6**, 1–11 (2021). 10.1136/bmjgh-2021-004993 PMC8336216 34344668

[7] Wondmieneh, A., Alemu, W., Tadele, N. & Demis, A. Medication administration errors and contributing factors among nurses: a cross-sectional study in tertiary hospitals, Addis Ababa, Ethiopia. *BMC Nurs.* **19**, 4 (2020). 10.1186/s12912-020-0397-0 PMC6958590 31956293

[8] Slawomirski, L. & Klazinga, N. The economics of patient safety: from analysis to action. (2020).

[9] Panagioti, M. et al. Prevalence, severity, and nature of preventable patient harm across medical care settings: systematic review and meta-analysis. 10.1136/bmj.l4185. (2019). 10.1136/bmj.l4185 PMC6939648 31315828

[10] Slawomirski, L., Auraaen, A. & Klazinga, N. The economics of patient safety in primary and ambulatory care: flying blind | OECD Health Working Papers | OECD iLibrary. https://www.oecd-ilibrary.org/social-issues-migration-health/the-economics-of-patient-safety-in-primary-and-ambulatory-care_baf425ad-en.

[11] Mekonnen, A. B., McLachlan, A. J. & Brien, J. A. E. Effectiveness of pharmacist-led medication reconciliation programmes on clinical outcomes at hospital transitions: a systematic review and meta-analysis. *BMJ Open* **6**, e010003 (2016). 10.1136/bmjopen-2015-010003 PMC4769405 26908524

[12] MacDowell, P., Cabri, A. & Davis, M. Medication administration errors. https://psnet.ahrq.gov/primer/medication-administration-errors (2021).

[13] Berdot, S. et al. Integration of a commercial barcode-assisted medication dispensing system in a teaching hospital. *Appl. Clin. Inform.* **10**, 615–624 (2019). 31434161 10.1055/s-0039-1694749 PMC6703993

[14] Rodziewicz, T. L., Houseman, B. & Hipskind, J. E. *Medical Error Reduction and Prevention*. (StatPearls Publishing, Treasure Island (FL), 2023).

[15] Mohamed Ibrahim, O., Ibrahim, R. M., Al Meslamani, A. Z. & Al Mazrouei, N. Dispensing errors in community pharmacies in the United Arab Emirates: investigating incidence, types, severity, and causes. *Pharm. Pract.* **18**, 1–8 (2020). 10.18549/PharmPract.2020.4.2111 PMC7603657 33149793

[16] Zirpe, K. G. et al. Incidence of medication error in critical care unit of a tertiary care hospital: where do we stand? *Indian J. Crit. Care Med.*10.5005/jp-journals-10071-23556 (2020) 10.5005/jp-journals-10071-23556 PMC7584841 33132563

[17] Berdot, S. et al. Evaluation of drug administration errors in a teaching hospital. *BMC Health Serv. Res* **12**, 60 (2012). 22409837 10.1186/1472-6963-12-60 PMC3364158

[18] Putriana, N., Nurjanah, N. & Kautsar, A. The relationship between medication errors in prescribing phase and service quality on national health insurance patients of pharmacy unit in public hospital in Bandung city. *Natl. J. Physiol. Pharm. Pharmacol.* **8**, 1 (2018).

[19] Roumeliotis, N. et al. Effect of electronic prescribing strategies on medication error and harm in hospital: a systematic review and meta-analysis. *J. Gen. Intern. Med.* **34**, 2210–2223 (2019). 31396810 10.1007/s11606-019-05236-8 PMC6816608

[20] Nuckols, T. K. et al. The effectiveness of computerized order entry at reducing preventable adverse drug events and medication errors in hospital settings: a systematic review and meta-analysis. *Syst. Rev.* **3**, 1–12 (2014). 24894078 10.1186/2046-4053-3-56 PMC4096499

[21] Gates, P. J., Hardie, R.-A., Raban, M. Z., Li, L. & Westbrook, J. I. How effective are electronic medication systems in reducing medication error rates and associated harm among hospital inpatients? A systematic review and meta-analysis. *J. Am. Med. Inform. Assoc.* **28**, 167–176 (2021). 33164058 10.1093/jamia/ocaa230 PMC7810459

[22] Baysari, M. T., Westbrook, J., Braithwaite, J. & Day, R. O. The role of computerized decision support in reducing errors in selecting medicines for prescription: narrative review. *Drug Saf.* **34**, 289–298 (2011). 21417501 10.2165/11588200-000000000-00000

[23] McCoy, A. B. et al. Real-time pharmacy surveillance and clinical decision support to reduce adverse drug events in acute kidney injury. *Appl. Clin. Inform.* **3**, 221–238 (2012). 22719796 10.4338/ACI-2012-03-RA-0009 PMC3377180

[24] Le Meur, Y. et al. Individualized mycophenolate mofetil dosing based on drug exposure significantly improves patient outcomes after renal transplantation. *Am. J. Transplant.* **7**, 2496–2503 (2007). 17908276 10.1111/j.1600-6143.2007.01983.x

[25] Newton, C. A. et al. A comparison study of continuous insulin infusion protocols in the medical intensive care unit: Computer-guided vs. standard column-based algorithms. *J. Hosp. Med.* **5**, 432–437 (2010). 20945468 10.1002/jhm.816 PMC3733454

[26] Dumont, C. & Bourguignon, C. Effect of a computerized insulin dose calculator on the process of glycemic control. *Am. J. Crit. Care* **21**, 106–116 (2012). 22381987 10.4037/ajcc2012956

[27] Geurts, D. et al. Implementation of clinical decision support in young children with acute gastroenteritis: a randomized controlled trial at the emergency department. *Eur. J. Pediatrics* **176**, 173–181 (2017). 10.1007/s00431-016-2819-2 PMC5243872 27933399

[28] Ashburner, J. M. et al. Electronic physician notifications to improve guideline-based anticoagulation in atrial fibrillation: a randomized controlled trial. *J. Gen. Intern. Med.* **33**, 2070–2077 (2018). 30076573 10.1007/s11606-018-4612-6 PMC6258628

[29] van Stiphout, F. et al. Effects of training physicians in electronic prescribing in the outpatient setting on clinical, learning and behavioural outcomes: a cluster randomized trial. *Br. J. Clin. Pharmacol.* **84**, 1187–1197 (2018). 29399852 10.1111/bcp.13540 PMC5980599

[30] Adusumalli, S. et al. Effect of passive choice and active choice interventions in the electronic health record to cardiologists on statin prescribing: a cluster randomized clinical trial. *JAMA Cardiol.* **6**, 40–48 (2021). 33031534 10.1001/jamacardio.2020.4730 PMC7542520

[31] Westbrook, J. I. I. et al. Short- and long-term effects of an electronic medication management system on paediatric prescribing errors. *NPJ Digit. Med.* **5**, 179 (2022). 10.1038/s41746-022-00739-x PMC9747795 36513770

[32] Tamblyn, R. et al. A randomized trial of the effectiveness of on-demand versus computer-triggered drug decision support in primary care. *J. Am. Med. Inform. Assoc.* **15**, 430–438 (2008). 18436904 10.1197/jamia.M2606 PMC2442270

[33] Strom, B. L. et al. Unintended effects of a computerized physician order entry nearly hard-stop alert to prevent a drug interaction: a randomized controlled trial. *Arch. Intern. Med.* **170**, 1578–1583 (2010). 20876410 10.1001/archinternmed.2010.324

[34] Haigh, J. M. & Caringi, R. G. Automation vs. human intervention: what is the best mix for optimum system performance? A case study. *Int. J. Risk Assess. Manag.* **7**, 708–721 (2007).

[35] Falemban, A. H. Medication-related problems and their intervention in the geriatric population: a review of the literature. *Cureus* **15**, e44594 (2023). 10.7759/cureus.44594 PMC10545972 37795072

[36] Moore, A., Rahman, A. & Tipper, C. Providing individualised feedback to improve the rates of prescription errors. *Arch. Dis. Child.* **105**, A50–A51 (2020).

[37] Osmani, F., Arab-Zozani, M., Shahali, Z. & Lotfi, F. Evaluation of the effectiveness of electronic prescription in reducing medical and medical errors (systematic review study). *Ann. Pharm. Fr.* **81**, 433 (2023). 36513154 10.1016/j.pharma.2022.12.002 PMC9737496

[38] Evrenoglou, T., Metelli, S. & Chaimani, A. *Introduction to Meta-Analysis*. *Principles and Practice of Clinical Trials*10.1007/978-3-319-52636-2_287 (2022).

[39] Rodgers, S. et al. Scaling-up a pharmacist-led information technology intervention (PINCER) to reduce hazardous prescribing in general practices: multiple interrupted time series study. *PLoS Med.* **19**, 1–19 (2022). 10.1371/journal.pmed.1004133 PMC9718399 36383560

[40] Higgins, J. P. T. et al. Cochrane Handbook for Systematic Reviews of Interventions. Cochrane Handbook for Systematic Reviews of Interventions 10.1002/9781119536604 (2022).

[41] Yazdani, A. et al. Automated misspelling detection and correction in persian clinical text. *J. Digital Imaging* **33**, 555–562 (2020). 10.1007/s10278-019-00296-y PMC7256143 31823185

[42] Chen, C. Y., Chen, Y. L., Scholl, J., Yang, H. C. & Li, Y. C. (J. ack) Ability of machine-learning based clinical decision support system to reduce alert fatigue, wrong-drug errors, and alert users about look alike, sound alike medication. *Comput. Methods Prog. Biomed.* **243**, 107869 (2024). 10.1016/j.cmpb.2023.107869 37924770

[43] Dahmke, H. et al. Development and validation of a clinical decision support system to prevent anticoagulant duplications. *Int. J. Med. Inform.* **187**, 105446 (2024). 38669733 10.1016/j.ijmedinf.2024.105446

[44] Miao, S. et al. The construction and application of a clinical decision support system for cardiovascular diseases: multimodal data-driven development and validation study. *JMIR Med. Inform.* **13**, e63186 (2025). 40029975 10.2196/63186 PMC11892944

[45] Hohl, C. M. et al. Why clinicians don’t report adverse drug events: qualitative study. *JMIR Public Health Surveill.* **4**, 1–11 (2018). 10.2196/publichealth.9282 PMC5849794 29487041

[46] Franklin, B. D., O’Grady, K., Donyai, P., Jacklin, A. & Barber, N. The impact of a closed-loop electronic prescribing and administration system on prescribing errors, administration errors and staff time: a before-and-after study. *Qual. Saf. Health Care* **16**, 279–284 (2007). 17693676 10.1136/qshc.2006.019497 PMC2464943

[47] Harrer, M., Cuijpers, P., Furukawa, T. A. & Ebert, D. D. Doing meta-analysis in R: a hand-on guide. (2019).

[48] Page, M. J., Sterne, J. A. C., Higgins, J. P. T. & Egger, M. Investigating and dealing with publication bias and other reporting biases in meta-analyses of health research: a review. *Res. Synth. Methods* **12**, 248–259 (2021). 33166064 10.1002/jrsm.1468

[49] Ciapponi, A. et al. Reducing medication errors for adults in hospital settings. *Cochrane Database Syst. Rev*. 10.1002/14651858.cd009985.pub2 (2021). 10.1002/14651858.CD009985.pub2 PMC8614640 34822165

[50] Singh, G., Patel, R. H., Vaqar, S. & Boster, J. Root cause analysis and medical error prevention. *StatPearls* (2024).

[51] Thabane, L. et al. A tutorial on sensitivity analyses in clinical trials: the what, why, when and how. *BMC Med. Res. Methodol.* **13**, 92 (2013). 10.1186/1471-2288-13-92 PMC3720188 23855337

[52] Elliott, R. A., Ann Elliott, R., Camacho, E. & Jankovic, D. Economic analysis of the prevalence and clinical and economic burden of medication error in England. *BMJ Qual. Saf.* **30**, 96–105 (2021). 32527980 10.1136/bmjqs-2019-010206

[53] Page, M. J. et al. PRISMA 2020 explanation and elaboration: updated guidance and exemplars for reporting systematic reviews. *BMJ* **372**, n160 (2021). 10.1136/bmj.n160 PMC8005925 33781993

[54] Higgins, J.P.T. et al. *Cochrane Handbook for Systematic Reviews of Interventions*. *Cochrane Handbook for Systematic Reviews of Interventions*10.1002/9781119536604. (2019).

[55] Eldridge, S. et al. Revised Cochrane risk of bias tool for randomized trials (RoB 2.0) Additional considerations for cluster-randomized trials. *J. Clin. Epidemiol.* **126**, 37–44 (2016). 10.1016/j.jclinepi.2020.06.015 32562833

[56] Frampton, G. eoff et al. Principles and framework for assessing the risk of bias for studies included in comparative quantitative environmental systematic reviews. *Environ. Evid.* **11**, 12 (2022). 38264537 10.1186/s13750-022-00264-0 PMC10805236

[57] Andrade, C. understanding relative risk, odds ratio, and related terms: as simple as it can get. *J. Clin. Psychiatry* **76**, 21865 (2015). 10.4088/JCP.15f10150 26231012

[58] Bodmer, W. F. Understanding Statistics. *J. R. Stat. Soc. Ser. A* **148**, 69 (1985).

[59] Alansari, R. A. Brief communication. *Saudi Med. J.* **40**, 954–957 (2019). 31522225 10.15537/smj.2019.9.23831 PMC6790486

[60] Zhang, A. et al. A meta-analysis of the effectiveness of telemedicine in glycemic management among patients with type 2 diabetes in primary care. *Int. J. Environ. Res. Public Health* **19**, 4173 (2022). 35409853 10.3390/ijerph19074173 PMC8999008

[61] Diel, A. et al. A systematic review and meta analysis on digital mental health interventions in inpatient settings. *npj Digit. Med.* **7**, 253 (2024). 10.1038/s41746-024-01252-z PMC11408664 39289463

