# Supplementary material for: Meta-analysis of randomized controlled trials of electronic health interventions to reduce medication errors
Source: NPJ Digit Med. 2025 Dec 17;8:767. doi: 10.1038/s41746-025-02127-7 (PMC12715224; doi:10.1038/s41746-025-02127-7)
Supplement: Supplementary file 1 — Supplementary Information [file 41746_2025_2127_MOESM1_ESM.pdf]

SUPPLEMENTAL MATERIAL

Supplementary Table 1. Information Sources

Database	Coverage
EBSCO	1810 to present
PubMed	1967 to present
Embase	1974 to present
Web of Science	1991 to present

(a) coverage years of each database

Supplementary Table 2. Literature search strategy for EBSCO

Step	Search text	Articles
1	drug prescribing OR drug prescription OR drug prescriptions	109.359
2	e prescribing OR e prescription OR electronic prescribing OR electronic prescription OR electronic transmission of prescriptions	5.157
3	(drug administration error or drug administration errors) OR (inappropriate prescribing or inappropriate prescribings) OR (inappropriate prescription or inappropriate prescriptions) OR (prescription error or prescription errors or prescriptions errors) OR (prescribing errors or prescribing error) OR (medication error or medication errors) OR over prescribing OR potentially inappropriate medications OR wrong drug administration	48.811
4	computer order entry OR computer physician order entry OR computer provide order entry OR cpoe OR electronic order entry OR medical order entry system OR medication alert system	6.948
5	1 AND 2 AND 3 AND 4	154

Supplementary Table 3. Literature search strategy for PubMed

Step	Search text	Articles
1	("drug prescribing" or "drug prescription" or "drug prescriptions")	124.106
2	("e prescribing" or "e prescription*" or "electronic prescribing" or "electronic prescription*" or "electronic transmission of prescriptions")	38.556
3	("computer* order entr*" or "computer* physician order entr*" or	17.531

	"computer* provider order entr*" or "cpoe" or "electronic order entr*" or "medical order entry system*" or "medication alert system*")	
4	("drug administration error" or "drug administration errors" or "inappropriate prescribing" or "inappropriate prescribings" or "inappropriate prescription" or "inappropriate prescriptions" or "prescription error" or "prescription errors" or "prescriptions errors" or "prescribing errors" or "prescribing error" or "medication error" or "medication errors" or "over prescribing" or "potentially inappropriate medications" or "wrong drug administration")	61.215
5	1 AND 2 AND 3 AND 4	449

For PubMed, search terms included a combination of free-text terms and indexed vocabulary (MeSH terms) where applicable, such as “Medication Errors”[MeSH] and “Electronic Prescribing”[MeSH].

Supplementary Table 4. Literature search strategy for Embase

Step	Search text	Articles
1	“drug prescribing’ OR ‘drug prescription’ OR ‘drug prescriptions’	8.628
2	“e prescribing’ OR ‘e prescription*’ OR ‘electronic prescribing’ OR ‘electronic prescription*’ OR ‘electronic transmission of prescriptions’	5.188
3	‘computer* order entr*’ OR ‘computer* physician order entr*’ OR ‘provider order entr*’ OR ‘cpoe’ OR ‘electronic order entr*’ OR ‘medical order entry system*’ OR ‘medication alert system*’	4.049
4	‘drug administration error’ OR ‘drug administration errors’ OR ‘inappropriate prescribing’ OR 'inappropriate prescribings’ OR 'inappropriate prescription’ OR 'inappropriate prescriptions’ OR ‘prescription error’ OR ‘prescription errors’ OR ‘prescriptions errors’ OR ‘prescribing errors’ OR ‘prescribing error’ OR ‘medication error’ OR ‘medication errors’ OR ‘over prescribing’ OR ‘potentially inappropriate medications’ OR ‘wrong drug administration’	33.410
5	1 AND 2 AND 3 AND 4	15

Supplementary Table 5. Literature search strategy for Web of Science

Step	Search text	Articles
1	((ALL=(‘drug prescribing’)) OR ALL=(‘drug prescription’)) OR ALL=(‘drug prescription’)	95.146
2	(((((ALL=(e prescribing)) OR ALL=(e prescription*)) OR ALL=(electronic prescribing)) OR ALL=(electronic prescription*)) OR ALL=(electronic transmission of prescriptions)	91.865
3	((((((((((((((ALL=(drug administration error)) OR ALL=(drug administration errors)) OR ALL=(inappropriate prescription)) OR ALL=(inappropriate prescriptions)) OR ALL=(prescription error) OR ALL=(prescription errors)) OR ALL=(prescriptions errors) OR ALL=(prescribing errors)) OR ALL=(prescribing error)) OR ALL=(medication errors)) OR ALL=(over prescribing)) OR ALL=(potentially inappropriate medications)) OR ALL=(wrong drug administration)	66.326
4	((((((ALL=(computer* order entr*)) OR ALL=(computer*physician order entr*)) OR ALL=(computer* provider order entr*)) OR ALL=(cpoe)) OR ALL=(electronic order entr*)) OR ALL=(medical order entry system*)) OR ALL=(medication alert system*)	16.659
5	1 AND 2 AND 3 AND 4	502

Supplementary Table 6. PRISMA 2020 Checklist

PRISMA 2020 Checklist

Section and Topic	Item #	Checklist item	Location where item is reported
**TITLE**			
Title	1	Identify the report as a systematic review.	Line 34
**ABSTRACT**			
Abstract	2	See the PRISMA 2020 for Abstracts checklist.	Line 29-43
**INTRODUCTION**			
Rationale	3	Describe the rationale for the review in the context of existing knowledge.	Line83-93
Objectives	4	Provide an explicit statement of the objective(s) or question(s) the review addresses.	Line 93-96
**METHODS**			
Eligibility criteria	5	Specify the inclusion and exclusion criteria for the review and how studies were grouped for the syntheses.	Line 334-338
Information sources	6	Specify all databases, registers, websites, organisations, reference lists and other sources searched or consulted to identify studies. Specify the date when each source was last searched or consulted.	Line 340-344
Search strategy	7	Present the full search strategies for all databases, registers and websites, including any filters and limits used.	Line 347-357
Selection process	8	Specify the methods used to decide whether a study met the inclusion criteria of the review, including how many reviewers screened each record and each report retrieved, whether they worked independently, and if applicable, details of automation tools used in the process.	Line 360-369
Data collection process	9	Specify the methods used to collect data from reports, including how many reviewers collected data from each report, whether they worked independently, any processes for obtaining or confirming data from study investigators, and if applicable, details of automation tools used in the process.	Line 371-383
Data items	10a	List and define all outcomes for which data were sought. Specify whether all results that were compatible with each outcome domain in each study were sought (e.g. for all measures, time points, analyses), and if not, the methods used to decide which results to collect.	Line 386-392
	10b	List and define all other variables for which data were sought (e.g. participant and intervention characteristics, funding sources). Describe any assumptions made about any missing or unclear information.	Line 392-397
Study risk of bias assessment	11	Specify the methods used to assess risk of bias in the included studies, including details of the tool(s) used, how many reviewers assessed each study and whether they worked independently, and if applicable, details of automation tools used in the process.	Line 400-409
Effect measures	12	Specify for each outcome the effect measure(s) (e.g. risk ratio, mean difference) used in the synthesis or presentation of results.	Line 412-422
Synthesis methods	13a	Describe the processes used to decide which studies were eligible for each synthesis (e.g. tabulating the study intervention characteristics and comparing against the planned groups for each synthesis (item #5)).	Line 425-427
	13b	Describe any methods required to prepare the data for presentation or synthesis, such as handling of missing summary statistics, or data conversions.	Line 428-434
	13c	Describe any methods used to tabulate or visually display results of individual studies and syntheses.	Line 436-441
	13d	Describe any methods used to synthesize results and provide a rationale for the choice(s). If meta-analysis was performed, describe the model(s), method(s) to identify the presence and extent of statistical heterogeneity, and software package(s) used.	Line 442-447
	13e	Describe any methods used to explore possible causes of heterogeneity among study results (e.g. subgroup analysis, meta-regression).	Line 448-449
	13f	Describe any sensitivity analyses conducted to assess robustness of the synthesized results.	Line 449-455
Reporting bias assessment	14	Describe any methods used to assess risk of bias due to missing results in a synthesis (arising from reporting biases).	Line 458-471
Certainty assessment	15	Describe any methods used to assess certainty (or confidence) in the body of evidence for an outcome.	Line 442-447
**RESULTS**			
Study selection	16a	Describe the results of the search and selection process, from the number of records identified in the search to the number of studies included in the review, ideally using a flow diagram.	Line 99-101
	16b	Cite studies that might appear to meet the inclusion criteria, but which were excluded, and explain why they were excluded.	Line 102-106
Study characteristics	17	Cite each included study and present its characteristics.	Line 111-120
Risk of bias in studies	18	Present assessments of risk of bias for each included study.	Line 122-127
Results of individual studies	19	For all outcomes, present, for each study: (a) summary statistics for each group (where appropriate) and (b) an effect estimate and its precision (e.g. confidence/credible interval), ideally using structured tables or plots.	Line 149-158
Results of syntheses	20a	For each synthesis, briefly summarise the characteristics and risk of bias among contributing studies.	Line 132-133
	20b	Present results of all statistical syntheses conducted. If meta-analysis was done, present for each the summary estimate and its precision (e.g. confidence/credible interval) and measures of statistical heterogeneity. If comparing groups, describe the direction of the effect.	Line 134-135
	20c	Present results of all investigations of possible causes of heterogeneity among study results.	Line 136-142
	20d	Present results of all sensitivity analyses conducted to assess the robustness of the synthesized results.	Line 160-181
Reporting biases	21	Present assessments of risk of bias due to missing results (arising from reporting biases) for each synthesis assessed.	Line 122-127
Certainty of evidence	22	Present assessments of certainty (or confidence) in the body of evidence for each outcome assessed.	Line 132-133
**DISCUSSION**			
Discussion	23a	Provide a general interpretation of the results in the context of other evidence.	Line 185-192
	23b	Discuss any limitations of the evidence included in the review.	Line 263-270
	23c	Discuss any limitations of the review processes used.	Line 218-225
	23d	Discuss implications of the results for practice, policy, and future research.	Line 280-300
**OTHER INFORMATION**			
Registration and protocol	24a	Provide registration information for the review, including register name and registration number, or state that the review was not registered.	Line 47
	24b	Indicate where the review protocol can be accessed, or state that a protocol was not prepared.	Line 47
	24c	Describe and explain any amendments to information provided at registration or in the protocol.	Line 47
Support	25	Describe sources of financial or non-financial support for the review, and the role of the funders or sponsors in the review.	Line 474-475
Competing interests	26	Declare any competing interests of review authors.	Line 491-492
Availability of data, code and other materials	27	Report which of the following are publicly available and where they can be found: template data collection forms; data extracted from included studies; data used for all analyses; analytic code; any other materials used in the review.	Line 476-484

Supplementary Table 7. PRISMA 2020 for Abstracts Checklist

PRISMA 2020 for Abstracts Checklist

Section and Topic	Item #	Checklist item	Reported (Yes/No)
TITLE			
Title	1	Identify the report as a systematic review.	Yes
BACKGROUND			
Objectives	2	Provide an explicit statement of the main objective(s) or question(s) the review addresses.	Yes
METHODS			
Eligibility criteria	3	Specify the inclusion and exclusion criteria for the review.	Yes
Information sources	4	Specify the information sources (e.g. databases, registers) used to identify studies and the date when each was last searched.	Yes
Risk of bias	5	Specify the methods used to assess risk of bias in the included studies.	Yes
Synthesis of results	6	Specify the methods used to present and synthesise results.	Yes
RESULTS			
Included studies	7	Give the total number of included studies and participants and summarise relevant characteristics of studies.	Yes
Synthesis of results	8	Present results for main outcomes, preferably indicating the number of included studies and participants for each. If meta-analysis was done, report the summary estimate and confidence/credible interval. If comparing groups, indicate the direction of the effect (i.e. which group is favoured).	Yes
DISCUSSION			
Limitations of evidence	9	Provide a brief summary of the limitations of the evidence included in the review (e.g. study risk of bias, inconsistency and imprecision).	No
Interpretation	10	Provide a general interpretation of the results and important implications.	Yes
OTHER			
Funding	11	Specify the primary source of funding for the review.	No
Registration	12	Provide the register name and registration number.	Yes

Supplementary Figure 1. Forest Plot of Subgroup Intervention

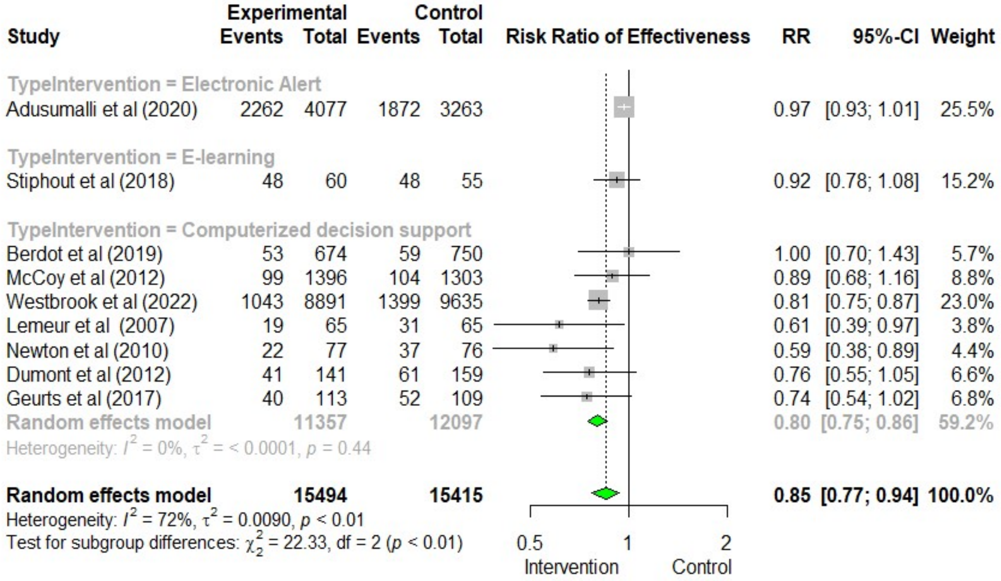

Supplementary Figure 2. Forest Plot of Subgroup Outcome

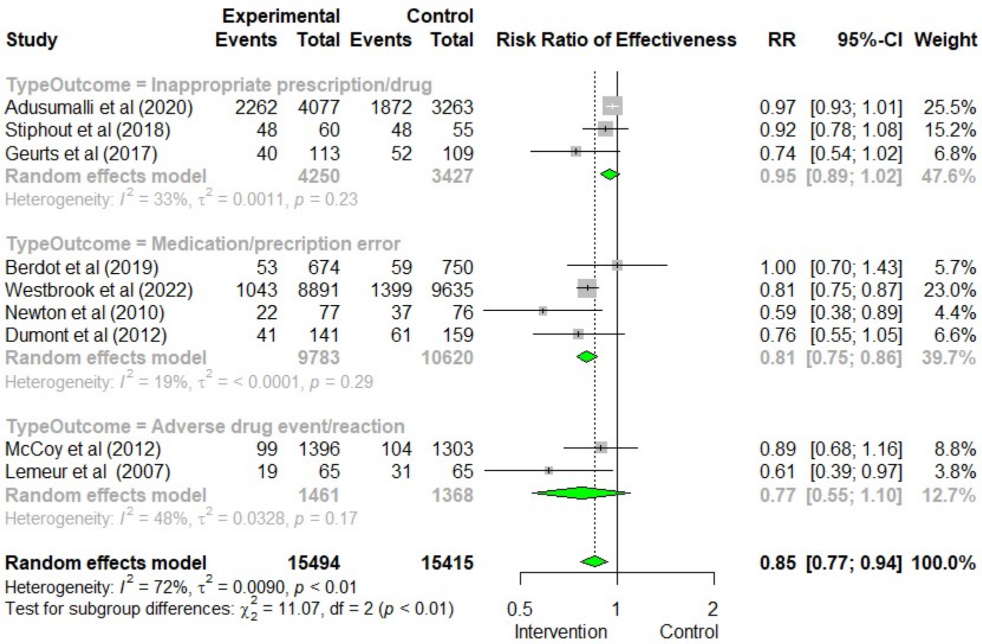

Supplementary Table 8. Population Characteristics

No	Author (year)	Gender		Age		Number of patient		Number of prescription		Number of physician		Number of medication		Number of care units
		Control	Intervention	Control	Intervention	Control	Intervention	Control	Intervention	Control	Intervention	Control	Intervention	
1	Lemeur et al (2007)	Female 42 Male 58	Female 29 Male 71	49 ± 13	50 ± 14	65	65							

No	Author (year)	Gender		Age		Number of patient		Number of prescription		Number of physician		Number of medication		Number of care units
		Control	Intervention	Control	Intervention	Control	Intervention	Control	Intervention	Control	Intervention	Control	Intervention	
2	Tamblyn et al (2008)	Female 949 Male 601	Female 1165 Male 734	67.3 (15.1)	66.9 (16)	1550	1899	51.9 (47.3)	50.8 (46.9)	Female 7 Male 7	Female 6 Male 8			
3	Newton et al (2010)	Female 46.7 Male 51.3	Female 42.9 Male 57.1	58.5 ± 13.4	57.8 ± 11.0	77	76							
4	Strom et al (2010)					44	52							
5	Dumont & Bourguignon (2012)	Female 59	Female 42	64.3 year (SD 10.3)	64.1 (12.1)	159	141							
6	McCoy et al (2012)	Female 39.2% Male 60.7%	Female 47% Male 53%	58.3 (15.7)	60.7 (16.8)	196	200					1303	1396	
7	Geurts et al (2017)	Male 57	Male 61	1.3 (0.8–2.4)	1.5 (0.9–2.4)	109	113					133 (92–184)	136 (98–206)	
8	Ashburner et al (2018)	Female 639 Male 725	Female 482 Male 490	76.3 (11.5)	75.7 (11.1)									

No	Author (year)	Gender		Age		Number of patient		Number of prescription		Number of physician		Number of medication		Number of care units
		**Control**	**Intervention**	**Control**	**Intervention**	**Control**	**Intervention**	**Control**	**Intervention**	**Control**	**Intervention**	**Control**	**Intervention**	
9	Stiphout et al (2018)	Female 265<br>Male 263	Female 289<br>Male 273	57 (17)	53 (15)	528	562			61	63	8.4 (4.3)	7.4(4.2)	
10	Berdot et al (2019)			18-60	18-60									6
11	Adusumalli et al (2020)	Female 1356<br>Male 1907	Passive<br>- Female 1840<br>- Male 2513<br>Active<br>- Female 1748<br>- Male 2329	63.6 (9.5)	Passive 63.8 (9.0)<br>Active 64 (9)	3263	Passive 4353<br>Active 4077			27	Passive 27<br>Active 28			
12	Westbrook et al (2022)	Female 691 (40.98)	70 days of eMM: 838 (39.98)<br>1 year post-eMM: 460 (44.27)"	73.9 months (62.4)	70 days of eMM: 88.4 months (63.4)<br>1 year post-eMM: 86.1 months (63.5)	1686	70 days of eMM: 2096<br>1 year post-eMM: 1039					9635 medication order	70 days of eMM: 16734<br>1 year post-eMM: 8891	

Supplementary Figure 3. Risk of Bias Domains

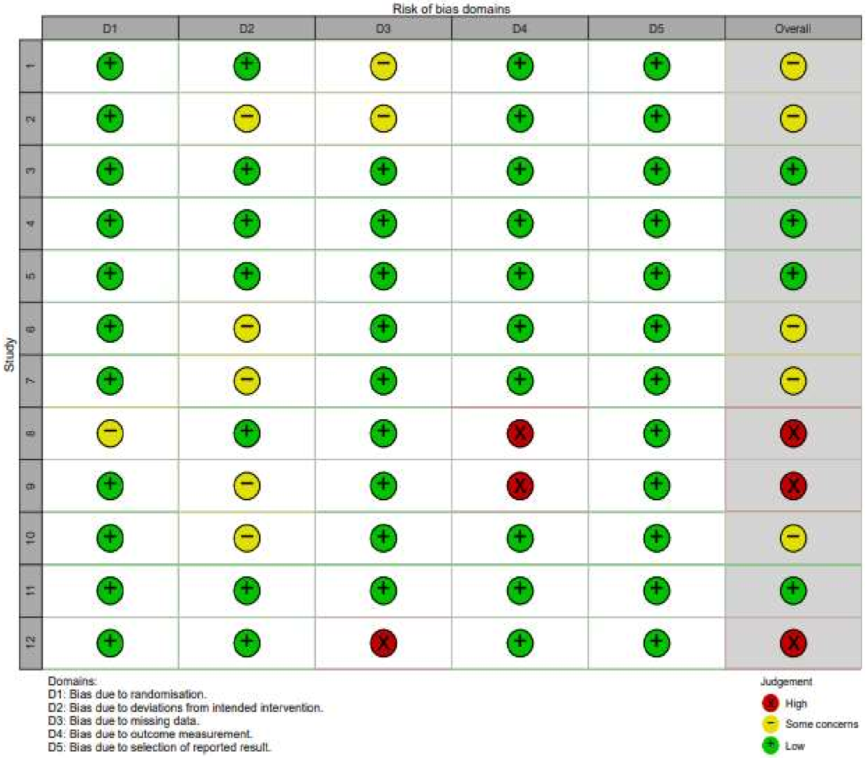

Supplementary Figure 4. Forest Plot All Included Studies

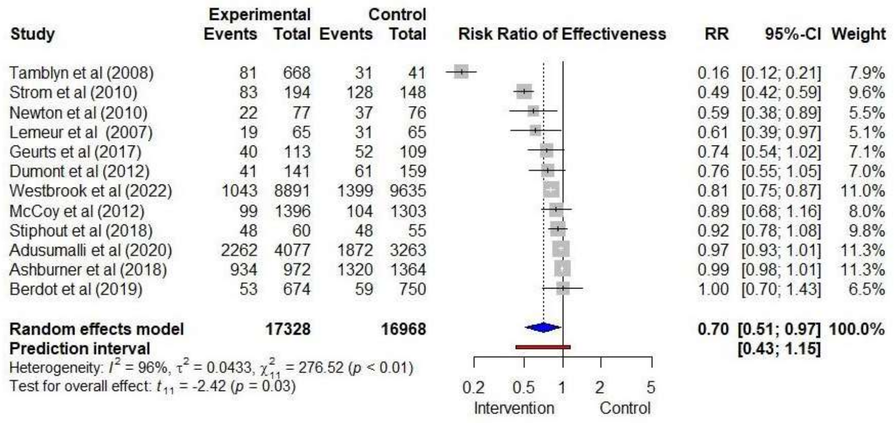

Supplementary Table 9. Basic Removal Outliers for Heterogeneity Test

Parameter	Value
Identified outliers (REML)	“1”, “2”, “4”
Random-Effects Model	(k=9; tau^2 estimator: REML)
tau^2 (estimated amount of total heterogeneity)	0.0408
Tau (square root of estimated tau^2 value)	0.2019
I^2 (total heterogeneity / total variability)	78.42%
H^2 (total variability / sampling variability)	4.63
Test for Heterogeneity	Q(df=8) = 36.4383<br>p-val < .0001
Model results	estimate -0.2927<br>se 0.0824<br>zval -3.5536<br>pval 0.0004<br>ci. lb -0.4542<br>ci.ub -0.1313
Signif. codes	0 ’***’ 0.001 ‘**’ 0.01 ‘*’ 0.05 ‘.’ 0,1 ‘ ‘ 1

Supplementary Table 10. Comparison before and after removal

Parameter	Before removal study	After removal study
RR	0.70	0.85
95%CI	[0.51 - 0.97]	[0.77 - 0.94]
Prediction interval	[0.43 - 1.15]	[0.66 - 1.09]
Random effects model	Experimental : 17328<br>Control : 16968	Experimental : 15494<br>Control : 15415
Weight	100%	100%
Heterogeneity	$I^2 = 96\%$<br>$\tau^2 = 0.0433$<br>$x^2_{11} = 276.52$ ($p < 0.01$)	$I^2 = 72\%$<br>$\tau^2 = 0.0090$<br>$x^2_{11} = 28.27$ ($p < 0.01$)
Test for overall effect	$t_{11} = -2.42$ ($p = 0.03$)	$z = -3.30$ ($p < 0.01$)

Supplementary Figure 5. LOU ES of Influence Analysis

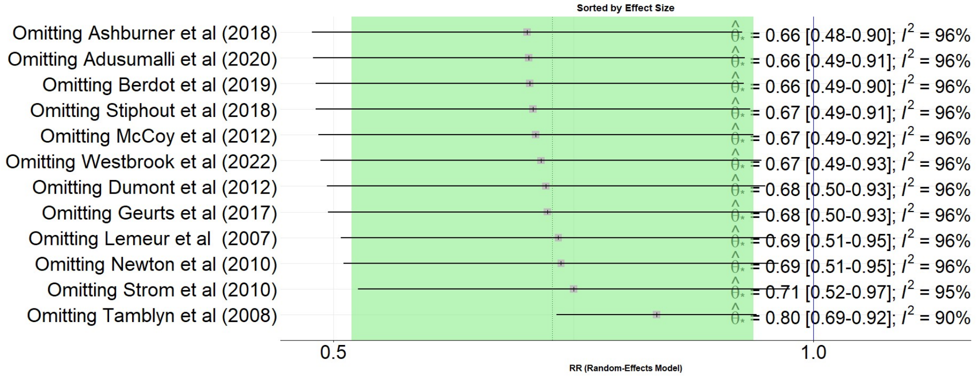

Supplementary Figure 6. LOU I2 of Influence Analysis

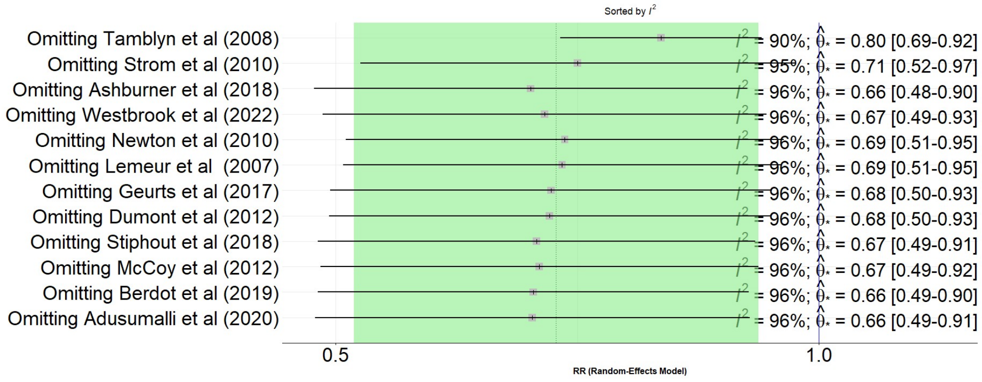

Supplementary Figure 7. IA of Influence Analysis

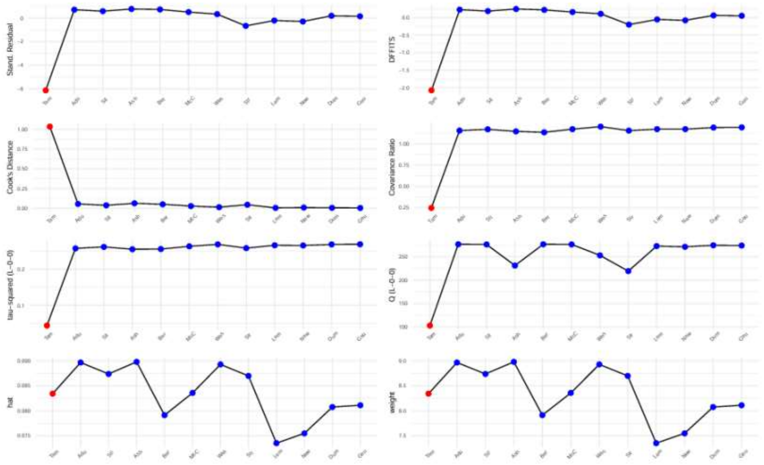

Supplementary Figure 8. Baujat of Influence Analysis

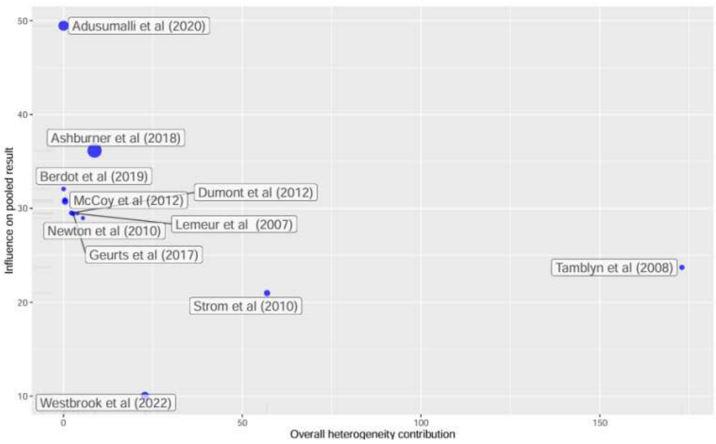

Supplementary Figure 9. GOSH Plot

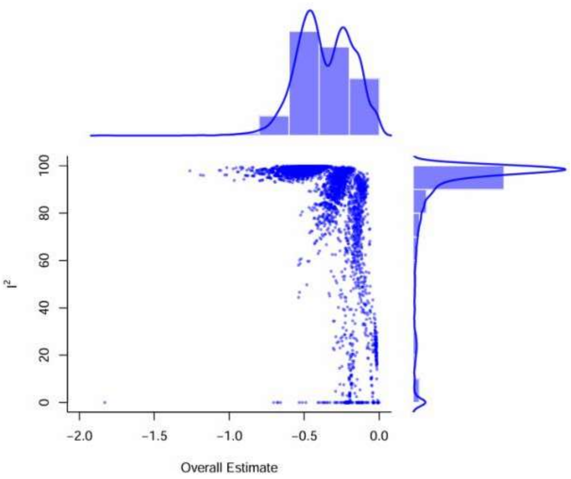

Supplementary Figure 10. Kmeans of GOSH Diag

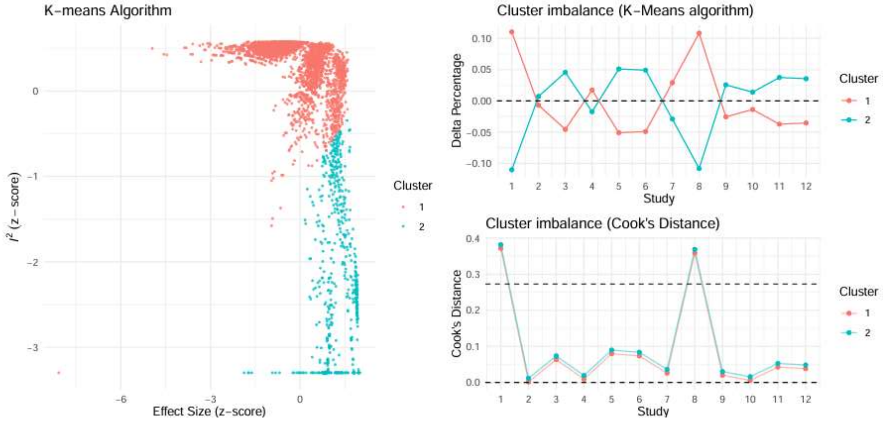

Supplementary Figure 11. DBSCAN of GOSH Diag

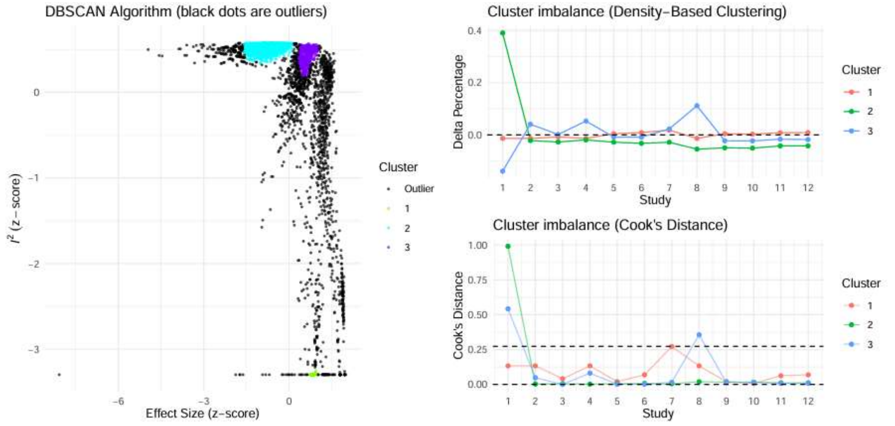

Supplementary Figure 12. GMM of GOSH Diag

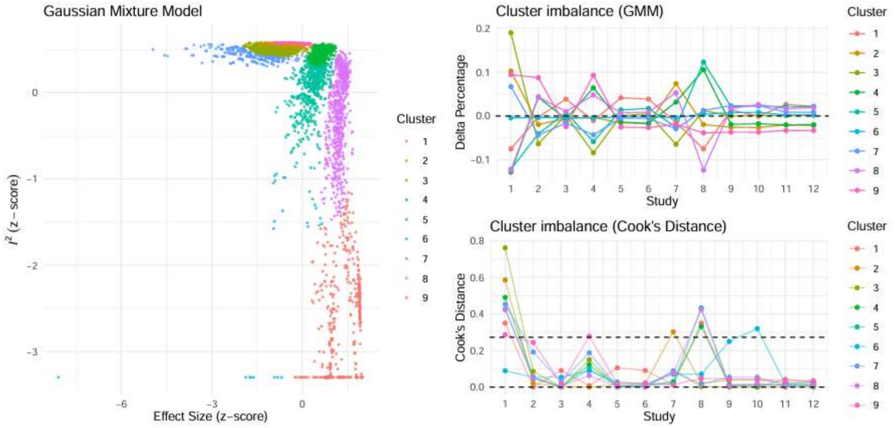

Supplementary Figure 13. Study 1

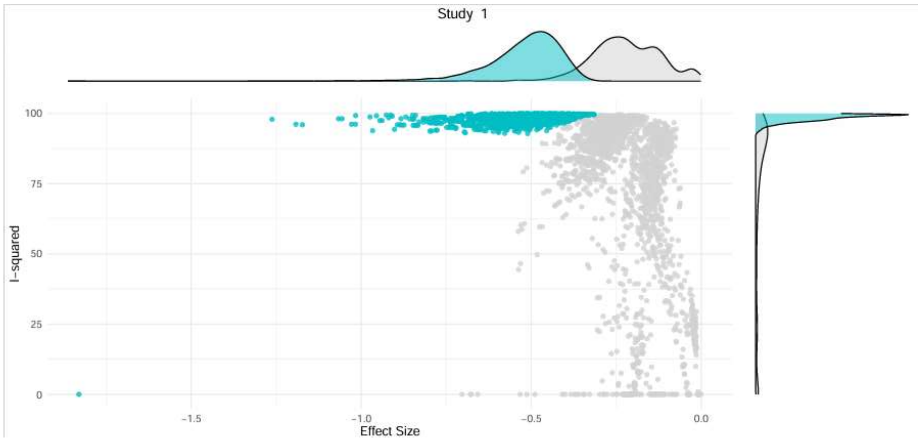

Supplementary Figure 14. Study 4

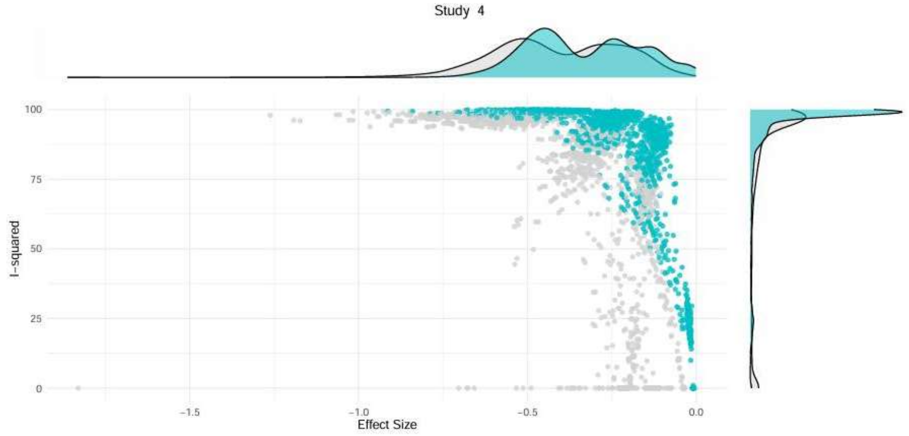

Supplementary Figure 15. Study 7

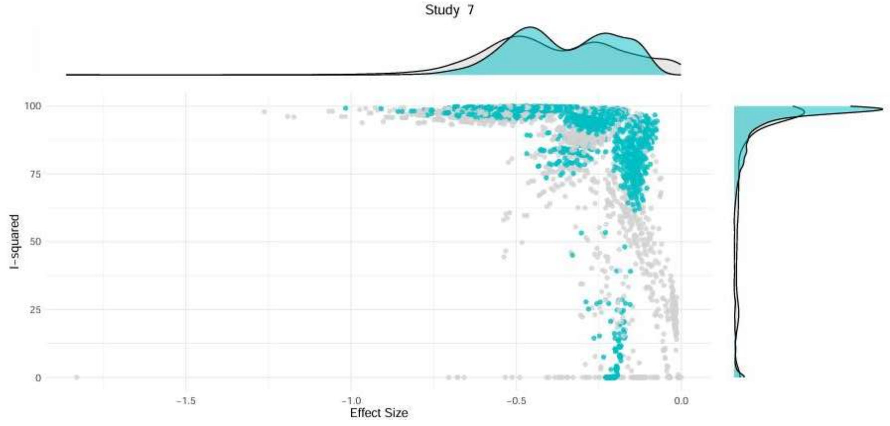

Supplementary Figure 16. Study 8

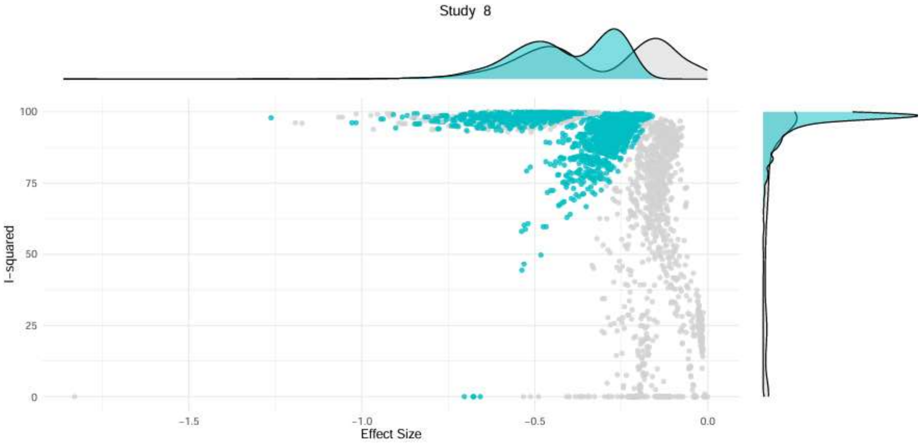

Supplementary Figure 17. Study 10

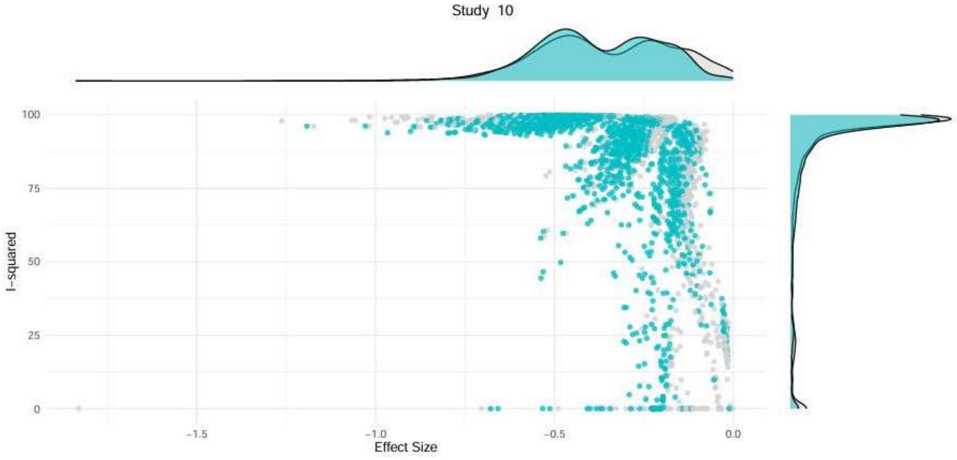

Supplementary Figure 18. Plot Cop

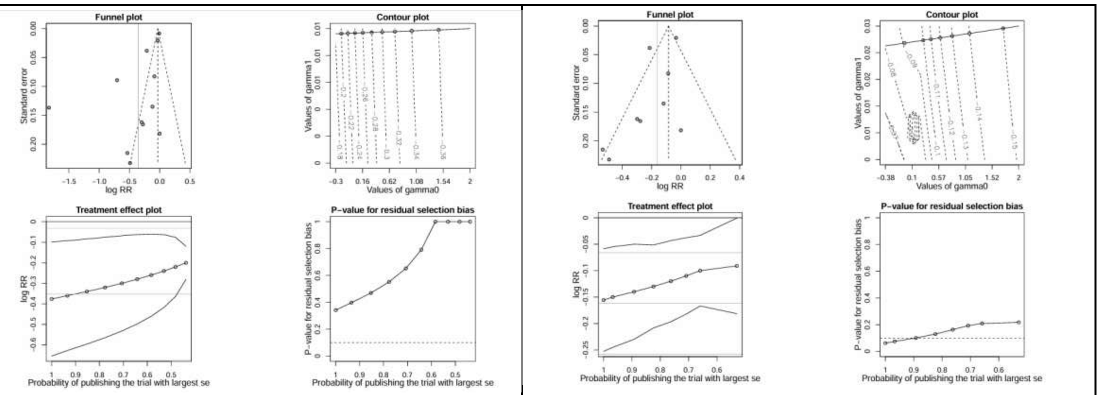

All studies

Post Removal Studies

Supplementary Table 11. Sensitivity Analysis Before Removal

	p.publ	RR	95%CI	tau^2	tau	p.trt	p.rsb	N
	1.0000	0.6864	[0.5198; 0.9063]	0.2223	0.4715	0.0079	0.3418	0
	0.9343	0.6976	[0.5341; 0.9113]	0.2163	0.4651	0.0083	0.3719	1
	0.8526	0.7118	[0.5510; 0.9195]	0.2109	0.4592	0.0092	0.4177	2
	0.7769	0.7261	[0.5685; 0.9273]	0.2061	0.4540	0.0103	0.4686	3
	0.7064	0.7408	[0.5873; 0.9343]	0.2019	0.4493	0.0113	0.5249	4
	0.6421	0.7557	[0.6079; 0.9396]	0.1981	0.4451	0.0117	0.5857	5
	0.5834	0.7711	[0.6312; 0.9420]	0.1948	0.4413	0.0109	0.6510	7
	0.5304	0.7866	[0.6588; 0.9393]	0.1919	0.4380	0.0080	0.7196	9
	0.4821	0.8025	[0.6949; 0.9268]	0.1894	0.4353	0.0027	0.7917	11
	0.4381	0.8188	[0.7554; 0.8875]	0.1873	0.4328	<0.0001	0.8666	13
Adjusted estimate		0.6864	[0.5198; 0.9063]	0.2223	0.4715	0.0079	0.3418	0
Unadjusted estimate		0.7023	[0.5091; 0.9688]	0.0433	0.2082	0.0341		

Supplementary Table 12. Sensitivity Analysis After Removal

	p.publ	RR	95%CI	tau^2	tau	p.trt	p.rsb	N
	1.0000	0.8559	[0.7767; 0.9430]	0.0067	0.0821	0.0017	0.0612	0
	0.9675	0.8608	[0.7862; 0.9468]	0.0062	0.0789	0.0020	0.0783	0
	0.8925	0.8694	[0.7946; 0.9513]	0.0054	0.0736	0.0023	0.1285	1
	0.8236	0.8782	[0.8119; 0.9499]	0.0047	0.0689	0.0012	0.1981	1
	0.7625	0.8870	[0.8215; 0.9578]	0.0042	0.0649	0.0022	0.2836	2
	0.7083	0.8959	[0.8335; 0.9631]	0.0039	0.0621	0.0029	0.3719	3
	0.6592	0.9049	[0.8464; 0.9675]	0.0037	0.0607	0.0034	0.4376	4
	0.5298	0.9130	[0.8340; 0.9995]	0.0041	0.0641	0.0488	0.5169	6
Adjusted estimate		0.8694	[0.7946; 0.9513]	0.0054	0.0736	0.0023	0.1285	1
Unadjusted estimate		0.8504	[0.7724; 0.9363]	0.0090	0.0949	0.0010		
